# Beyond Sarcopenia: older adults with type II diabetes mellitus tend to experience an elevated risk of poor dynamic balance—a case–control study

**DOI:** 10.1186/s12877-022-02826-w

**Published:** 2022-02-18

**Authors:** Chih-Chun Lin, Horng-Yih Ou, Hsiu-Yun Hsu, Kai-Pi Cheng, Tai-Jung Hsieh, Chien-Hsien Yeh, Fong-Chin Su, Li-Chieh Kuo

**Affiliations:** 1 Medical Device Innovation Center, National Cheng Kung University, Tainan, Taiwan; 2 Department of Internal Medicine, College of Medicine, National Cheng Kung University, Tainan, Taiwan; 3 Department of Occupational Therapy, College of Medicine, National Cheng Kung University, Tainan, Taiwan; 4 Department of Orthopedics, E-Da Hospital, Kaohsiung, Taiwan; 5 Department of Biomedical Engineering, College of Engineering, National Cheng Kung University, Tainan, Taiwan

**Keywords:** Diabetes Mellitus, Sarcopenia, Frailty, Senior Fitness Test, BMI, Dynamic Balance

## Abstract

**Background:**

People with type 2 diabetes mellitus (T2DM) tend to be vulnerable to geriatric syndromes such as sarcopenia and frailty. Reduced physical activity also accompanies sarcopenia and frailty, which is generally typical of patients with T2DM. However, a comprehensive assessment of physical fitness in patients with T2DM has seldom been carried out and verified. This study is thus an attempt to determine the associations among sarcopenia, frailty, and the SFT in diabetic patients and non-diabetic controls to provide a more comprehensive understanding of such associations in future evaluations of T2DM in older individuals.

**Methods:**

Sarcopenia, frailty, and the senior fitness test (SFT) were compared between 78 older men with T2DM (66.5 ± 9.0 years) and 48 age-matched normoglycemic controls (65.8 ± 5.3 years) in this case–control study. The skeletal muscle index (SMI), grip strength, and 4-m walk test were employed to assess for sarcopenia. Frailty was evaluated using the Study of Osteoporotic Fractures index (SOF). The SFT comprises five components, including body composition, muscle strength, flexibility, balance, and aerobic endurance.

**Results:**

The risk level of sarcopenia was significantly higher (*p* < 0.05) in the T2DM group as compared to the control group. No significant difference between-group differences were found in SMI and grip strength in the T2DM and control groups. However, the T2DM group showed a significant decrease in gait speed (*p* < 0.01) in comparison with the control group, as well as significant increases in frailty (*p* < 0.01) and depression (*p* < 0.05). With respect to the SFT, obvious elevation in BMI, significant declines in extremity muscle strength (elbow extensor, knee flexor, hip abductor, hip flexor, sit to stand), static/dynamic balance (single leg stand: *p* < 0.05; up-and-go: *p* < 0.01) and aerobic endurance (2-min step: *p* < 0.01; 6-min walk: p < 0.01) were found in the T2DM group. Furthermore, the SOF (OR = 2.638, 95% CI = 1.333–5.221), BMI (OR = 1.193, 95% CI = 1.041–1.368) and up-and-go (OR = 2.089, 95% CI = 1.400–3.117) were found to be positively and significantly associated with T2DM.

**Conclusions:**

The findings of this study indicated the importance of countering frailty and maintaining physical fitness, especially dynamic balance, during the early physical deterioration taking place in diabetic patients.

## Background

Diabetes mellitus (DM) is a global epidemic disease afflicting nearly 500 million people worldwide, and as the population ages, the number is projected to reach 700 million by 2045 [1]. With socio-economic growth and lifestyle changes, Asians have experienced a rapid increase in the prevalence of DM, which now accounts for 60% of the world's diabetic population [2], with the middle-aged and elderly being particularly susceptible. In the United States, the prevalence of DM has increased over the past two decades, during which time the prevalence of elderly people (> 65 y/o) has increased by 10%, and the prevalence of middle-aged adults (35–64 y/o) has increased by 3% [3]. In India, the prevalence of diabetes peaks at 60–69 years of age [4]. By comparison, in Taiwan, patients with diabetes over 65 years of age account for more than 50% of the total diabetic population [5]. As the prevalence of diabetes increases with age, older adults with DM tend to become vulnerable to geriatrics problems, such as sarcopenia and frailty.

Sarcopenia is an age-related syndrome characterized by a gradual loss of skeletal muscle mass and physical function [6]. A noticeable decline in muscle mass can be observed from nearly the fifth decade of life [7], with a loss of 1–2% per year [8]. The prevalence of sarcopenia increases steeply in an individual’s 60 s, with 5–13% of people aged 60 to 70, and 11–50% for people over 80 [6, 9]. Sarcopenia shares numerous characteristics with type 2 DM (T2DM), such as reduced physical activity, limited mobility, insulin resistance, chronic inflammation, and mitochondria dysfunction, among others [10–12]. Sarcopenia is also considered to be the underlying cause of physical frailty. In response, the impact of frailty on diabetes and its treatment has gradually attracted attention in recent years [13, 14]. The phenotype of frailty is defined as having five components, i.e., exhaustion, physical inactivity, muscle weakness, slow walking speed, and weight loss [15]. Among them, muscle weakness and slow walking speed are also two features of sarcopenia. Sarcopenia and frailty are considered to share similar pathological features in older adults, and T2DM may accelerate their progression [12, 16]. Previous research works on the physical inactivity of patients with diabetic mainly focused on the limitations of activities of daily living (ADL), and instrumental activities of daily living (IADL) [17–19], while investigation on functional limitations mainly focused on the evaluation of transfer and locomotion such as rising from chair, walking, and climbing 10 steps [20–22]. Some of the studies have discussed the effects of diabetes on grip strength [23] and gait speed [24, 25], the two screening criteria of sarcopenia; however, a comprehensive assessment of physical fitness in T2DM has seldom been clarified The senior fitness test (SFT), first proposed by Rikli and Jones, is now frequently used to assess physical performance in older adults [26]. The SFT measures five elements of physical performance, including body composition, muscle strength, flexibility, balance, and aerobic endurance, to evaluate the physiologic reserve of seniors and to identify at-risk participants [27]. In this study, we investigated five specific elements of the physical performance of patients with T2DM and further explored the muscle strength of the upper and lower limbs, including the elbow flexors/extensors, knee flexors/extensors, hip flexors/extensors, and hip abductors, to investigate the potential effects of T2DM on these muscle groups.

Sarcopenia, frailty, and reduced physical capacity seem to be the three commonly-seen characteristics of T2DM from clinical observations. However, it is still not clear which of these factors are more profound in the clinical manifestations of T2DM. The present study thus attempted to comprehend the associations among sarcopenia, frailty and SFT in diabetic patients and non-diabetic controls to provide more prospects for further responses for T2DM in older age.

## Methods

### Experimental Approach to the Problem

Data collection was performed from August 2018 to November 2020. After explaining the research procedures and risks, participants provided written and informed consent to participate in this study which was approved by the Institutional Review Board under the Number A-ER-106–303 according to the Helsinki declaration.

### Participants

Participants with T2DM who are over 50 years old and have been regularly followed and treated at the Diabetes Outpatient Clinic of National Cheng Kung University Hospital were recruited. The diagnosis of type 2 diabetes was based on the 2016 American Diabetes Association criteria [28], or current treatment with any anti-diabetic agents. Meanwhile, the age-matched normoglycemic participants in this case–control study were recruited through advertisement by the medical center. The exclusion criteria included a recent history of neuromuscular diseases, limb trauma, skin infections, cognitive dysfunction, acute stroke, acute pancreatitis, acute infections, cancer, and pregnancy. From August 2018 to November 2020, 134 participants were invited to participate in the study, of which 127 agreed and signed the informed consent form after our explanation of the study purposes and procedures. One was then excluded due to personal reasons. The final cohort consisted of 126 patients (67 women), with a mean age of 65.7 ± 8.6 years. The control group consisted of 48 healthy individuals (26 women), with a mean age of 65.8 ± 5.3 years, while the T2DM group consisted of 78 diabetic individuals (41 women), with a mean age of 66.5 ± 9.0 years. The average HbA1c (%) of control group was 5.62 ± 0.27, while the HbA1c of the T2DM group was 6.88 ± 0.73. The collected descriptive data are summarized in Table 2. In order to reduce potential bias, all data on sarcopenia-related factors, frailty, depression, and the five-element SFT were collected by one well-trained clinical staff member. The procedure is described as follows.

### Procedures

#### Detection of sarcopenia

The Asian Working Group for Sarcopenia (AWGS) has defined the criteria of sarcopenia as low muscle mass plus low muscle strength or poor physical performance [6, 29]. The cut-off points of the AWGS 2014 and AWGS 2019 are shown in Table 1. The AWGS defines low muscle mass plus low muscle strength or gait speed as sarcopenia; it also defines low muscle mass combined with low muscle strength and gait speed as severe sarcopenia. AWGS 2019 also added the term possible sarcopenia,” which is defined as either low muscle strength or reduced physical performance. The European Working Group on Sarcopenia in Older People (EWGSOP) suggested conceptual stages of sarcopenia as “presarcopenia,” “sarcopenia,” and “severe sarcopenia,” which were identified as “low muscle mass without the other two criteria,” “low muscle mass with either low muscle strength or low physical performance,” and “met the three criteria,” respectively. In this study, we modified the classification and defined the three criteria as four levels of risk, namely sarcopenia risk-level, from 0 to 3, in which 0 means asymptomatic, while 1 to 3 means the number of criteria met. The muscle mass represented by the skeletal muscle index (SMI) was calculated by the appendicular skeletal muscle mass (ASM) divided by height squared (kg/m^2^). Appendicular skeletal muscle mass of left arm (LA), right arm (RA), left leg (LL) and right leg (RL) was detected using a bioelectrical impedance analysis (BIA; TANITA MC-780MA, Tanita Corporation, Tokyo, Japan), while grip strength was measured using handgrip dynamometers (microFET® HandGRIP dynamometers, Hoggan Scientific, Salt Lake City, UT). Meanwhile, the usual 4-m gait speed that represents physical performance was assessed with a stopwatch. In addition, the SARC-F test [30], which evaluates strength, assistance with walking, rise from a chair, climbing stairs, and falls, was also used as a questionnaire to evaluate sarcopenia. The SARC-F scores range from 0 to 10, with a score equal to or greater than 4 indicating sarcopenia.

**Table 1:** AWGS 2014 and AWGS 2019 cut-off points for sarcopenia

	**Cut-off points AWGS 2014**	**Cut-off points AWGS 2019**
**Skeletal muscle index (kg/m2)**	Male: < 7.0	Male: < 7.0
	Female: < 5.7	Female: < 5.7
**Grip strength (kg)**	Male: < 26	Male: < 28
	Female: < 18	Female: < 18
**Gait speed (m/s)**	0.8	1

#### Frailty and depression

Frailty was detected using the Study of Osteoporotic Fractures index (SOF) [31–33], which provides a plain assessment of frailty in clinical practice. The SOF queries weight loss, lower extremity function, and energy perception via scores from 0 to 3, with a total score of 2 or 3 meaning frail, 1 indicating pre-frail, and 0 representing robust. To investigate depression, a modified version of the Patient Health Questionnaire (PHQ)-2 [34] was employed, in which the original 0 to 3 point-scoring method was revised to 0 (no) and 1 (yes). This modification was based on a suggestion by the Ministry of Health and Welfare of the Taiwan Government. The SOF and modified PHQ2 were used to detect frailty and depression of the participants in this study because these two assessments have been widely used for applications in long-term care services by case managers or care specialists in Taiwan.

#### Senior fitness test

The reliability of the SFT has been well demonstrated [35, 36], and its evaluation in this study was performed as previously described [27, 37, 38]. Body composition was evaluated using the body mass index (BMI), which was calculated as the ratio of an individual's weight divided by their height squared (kg/m^2^). Muscle strength of the lower extremity was evaluated by the 30-s sit-to-stand (STS) test. Participants were instructed to rise as fast as possible from a seat in 30 s with arms folded across the chest. The number of full stands in 30 s was recorded. In addition, we also measured the muscle strength of the elbow flexors/extensors, knee flexors/extensors, hip flexors/extensors, and hip abductors with a hand-held dynamometer (microFET®2, Hoggan Scientific, Salt Lake City, UT), as previously described [26, 39]. In the assessment, the participants performed three trials of maximum voluntary isometric contraction for each muscle. The mean of three trials was used to determine the strength of the particular muscle. Flexibility of the upper body was assessed by a back stretch. Participants were asked to reach over the shoulder with one hand and extend the other one up the middle of the back. The distance (cm) between the extended middle fingers between both hands was then recorded. Flexibility of the lower body was assessed using the sit-and-reach test. Participants sat on the edge of a chair, stretched out one leg, hands overlapped, and reached towards the toes. The distance between fingers and feet was recorded. Static balance was assessed using a test involving standing on one leg with the eyes open. Participants were asked to stand on one foot and maintain balance, the total time of which was recorded, with those exceeding 30 s being recorded as 30 s. Dynamic balance was assessed using the 8-foot up-and-go test. Participants were instructed to get up from a seat, walk 8 feet, turn, and return to the seated position. The time to complete this task was recorded. Aerobic endurance was assessed using the 2-min step test and 6-min walk test (6MWT). In the 2-min step test, the participants were instructed to raise each knee to a point midway between the patella and iliac crest in 2 min. The score was the number of times the right knee reached the targeted height. In the 6MWT, the participants were asked to walk vigorously for 6 min in a 30-m-long quiet corridor. The total distance that the participants walked during the time period was recorded.

### Statistical Analyses

Data are presented as mean ± standard deviation (SD). All statistical analyses were performed with Statistical Package for the Social Sciences (SPSS) software, version 17.0 (SPSS Inc, Chicago, IL). Comparisons of the sarcopenia, frailty, depression, and physical fitness between healthy controls and participants with type 2 diabetes were carried out using an independent samples t-test. The factors with significant differences in the t-test were chosen for subsequent univariate logistic analysis. Covariates that showed significance (*p* < 0.05) in the univariate analysis were entered in a stepwise forward multivariate logistic regression model. The odds ratios (OR) with 95% confidence intervals (95%CI) were also further presented to indicate the strength of association between diabetes and the selected factors. *P*-values below 0.05 were considered statistically significant.

## Results

### Sarcopenia, frailty, and depression in healthy individuals and participants with T2DM

The control group consisted of 48 healthy individuals (26 women), with a mean age of 65.8 ± 5.3 years, while the T2DM group consisted of 78 diabetic individuals (41 women), with a mean age of 66.5 ± 9.0 years. There were no significant between-group differences in the control and T2DM groups in terms of demographic information, including age, gender, height, and weight (Table 2). There were no significant differences between the two groups in terms of comorbidities and living habits such as smoking and drinking. Similarly, there were no significant differences in SMI, ASM, and grip strength between the control and T2DM groups; however, the gait speed was significantly decreased in the T2DM group. In addition, the sarcopenia risk-level was significantly higher in the T2DM group, as evaluated using either the AWGS 2014 or AWGS 2019. The proportion of participants suffering from sarcopenia in the T2DM group was higher than that in the control group in both the AWGS 2014 and AWGS 2019, but lacked statistical significance. Further, the number of people with possible sarcopenia was significantly higher in the diabetic participants compared with the controls. Lastly, although there was no significant difference in SARC-F between the control and T2DM groups, frailty and depression were significantly higher in the T2DM group.

**Table 2:** Characteristics and the diagnosis of sarcopenia, frailty and depression in the control and T2DM groups

	**Control** **(*N* = 48)**	**T2DM** **(*N* = 78)**	***p*-value**
**Descriptive Data**			
Age (y/o)	65.8 ± 5.3	66.5 ± 9.0	0.558
Gender			0.862
Male (n (%))	22 (45.8)	37 (47.4)	
Female (n (%))	26 (54.2)	41 (52.6)	
Height (cm)	160.4 ± 8.2	159.7 ± 7.7	0.622
Weight (kg)	62.3 ± 10.9	65.7 ± 10.9	0.092
Hypertension	16 (33.3)	39 (50.0)	0.068
Alcoholism	2 (4.2)	5 (6.4)	0.597
Smoking	0 (0)	1 (1.3)	0.435
Oral antidiabetic agents	-	66 (84.6)	
Insulin injection	-	20 (25.6)	
**Evaluation of Sarcopenia**			
SMI (kg/m2)	6.98 ± 1.10	7.28 ± 1.21	0.160
ASM (kg)			
LA	2.06 ± 0.53	2.21 ± 0.58	0.131
RA	2.13 ± 0.54	2.27 ± 0.61	0.195
LL	6.98 ± 1.57	7.06 ± 1.70	0.794
RL	7.01 ± 1.62	7.11 ± 1.72	0.721
Grip (non-dominant) (kg)	24.20 ± 8.14	22.25 ± 7.99	0.188
Grip (dominant) (kg)	25.19 ± 8.57	22.59 ± 8.55	0.102
4-m walk (m/s)	1.59 ± 0.32	1.40 ± 0.38	0.004*
Sarcopenia Risk Level 2014	0.42 ± 0.61	0.68 ± 0.77	0.029*
Sarcopenia Risk Level 2019	0.44 ± 0.62	0.81 ± 0.86	0.004*
AWGS 2014 Sarcopenia (n (%))	3 (6.3)	11 (14.1)	0.208
AWGS 2019 Sarcopenia (n (%))	3 (6.3)	12 (15.4)	0.208
AWGS 2019 possible sarcopenia (n (%))	16 (32)	41 (52.6)	0.047*
SARCF	0.42 ± 0.82	0.60 ± 0.92	0.269
**Frailty and Depression**			
SOF	0.21 ± 0.82	0.60 ± 0.92	0.004*
PHQ-2	0.06 ± 0.32	0.28 ± 0.64	0.012*

*ASM* Appendicular skeletal muscle mass, *LA* Left arm muscle mass, *RA* Right arm muscle mass, *LL* Left leg muscle mass, *RL* Right leg muscle mass

^*^ indicates statistically significant

### Physical Fitness in healthy individuals and participants with T2DM

As shown in Table 3, BMI was obviously higher in the T2DM group. Muscle strength was significantly reduced in the T2DM group, which predominately manifested in the elbow extensor, knee flexor, hip abductor, hip flexor, and STS test. There were no significant differences in flexibility between the control and T2DM groups. Further, static/dynamic balance and aerobic endurance were significantly poorer in the T2DM group than in the controls.

**Table 3:** Physical Fitness between the control and T2DM groups

	**Control** **(*N* = 48)**	**T2DM** **(*N* = 78)**	***p*-value**
**Body Composition**			
BMI (kg/m2)	24.10 ± 3.15	25.73 ± 3.53	0.010*
**Muscle Strength**			
Elbow flexor (non-dominant) (kg)	17.25 ± 5.67	15.92 ± 6.63	0.250
Elbow flexor (dominant) (kg)	17.23 ± 5.91	16.29 ± 6.98	0.436
Elbow extensor (non-dominant) (kg)	9.82 ± 2.58	8.92 ± 3.22	0.105
Elbow extensor (dominant) (kg)	9.69 ± 2.52	8.52 ± 2.78	0.019*
Knee flexor (L) (kg)	13.45 ± 5.52	11.11 ± 5.05	0.016*
Knee flexor (R) (kg)	14.16 ± 5.14	11.64 ± 5.13	0.009*
Knee extensor (L) (kg)	19.65 ± 5.42	17.87 ± 8.23	0.145
Knee extensor (R) (kg)	19.08 ± 6.33	18.66 ± 9.20	0.768
Hip abductor (L) (kg)	21.28 ± 11.20	17.67 ± 6.71	0.026*
Hip abductor (R) (kg)	20.14 ± 6.35	18.50 ± 7.27	0.201
Hip flexor (L) (kg)	16.54 ± 4.87	14.30 ± 6.11	0.025*
Hip flexor (R) (kg)	17.96 ± 7.30	15.06 ± 6.10	0.018*
Hip extensor (L) (kg)	14.82 ± 5.54	13.87 ± 6.38	0.402
Hip extensor (R) (kg)	15.16 ± 5.95	14.17 ± 6.99	0.421
Sit to stand (STS) (no. of stands)	18.58 ± 5.32	15.08 ± 5.15	< 0.001*
**Flexibility**			
Back stretch (cm)	-4.86 ± 13.17	-9.27 ± 13.54	0.076
Sit reach (L) (cm)	4.27 ± 12.58	0.68 ± 10.94	0.095
Sit reach (R) (cm)	4.52 ± 12.92	0.53 ± 11.79	0.079
**Balance**			
Single leg stand (s)	22.26 ± 10.26	15.32 ± 10.61	< 0.001*
Up-and-go (s)	5.45 ± 0.90	7.21 ± 2.77	< 0.001*
**Aerobic Endurance**			
2-min step (no. of steps)	101.31 ± 19.25	87.61 ± 20.21	< 0.001*
6-min walk (m)	531.46 ± 66.50	458.76 ± 103.25	< 0.001*

^*^ indicates statistically significant

### Dynamic balance and frailty are associated with T2DM

The results of univariate and multivariate analysis of covariates potentially related to T2DM are presented in Table 4. The SOF, BMI and up-and-go were found to be positively and significantly associated with T2DM, with an OR and 95% CI of 2.638 (1.333–5.221), 1.193 (1.041–1.368) and 2.089 (1.400–3.117), respectively.

**Table 4:** Diabetes-related factors in this study

	**Univariate logistic regression**			**Multivariate logistic regression**			
	**OR**	**95% CI**	**p-value**		**OR**	**95% CI**	***p*-value**
4-m walk	0.218	0.073–0.654	0.007*				
Sarcopenia Risk Level 2014	1.761	1.020–3.040	0.042*				
Sarcopenia Risk Level 2019	1.962	1.170–3.288	0.011*				
AWGS possible sarcopenia	0.475	0.225–1.002	0.051				
SOF	2.244	1.200–4.194	0.011*		2.638	1.333–5.221	0.005*
PHQ-2	2.744	0.999–7.541	0.05				
BMI	1.157	1.032–1.298	0.013*		1.193	1.041–1.368	0.011*
Elbow Extensor	0.866	0.755–0.994	0.041*				
Knee Flexor	0.909	0.844–0.980	0.012*				
Hip Abductor	0.951	0.904–1.002	0.058				
Hip Flexor	0.926	0.869–0.988	0.019*				
Sit to stand	0.880	0.816–0.949	0.001*				
Single leg stand	0.939	0.906–0.974	0.001*				
Up and go	1.964	1.366–2.824	< 0.001*		2.089	1.400–3.117	< 0.001*
2-min step	0.963	0.942–0.984	0.001*				
6-min walk	0.99	0.985–0.995	< 0.001*				

*OR* Odds ratio, *CI* Confidence interval

^*^ indicates statistically significant

## Discussion

In this study, we compared several diabetic-related conditions, including sarcopenia, frailty, and physical fitness in healthy controls and patients with T2DM in older adults. Regarding the diagnoses of sarcopenia, the criteria vary slightly among associations. In 2019, the AWGS released a consensus update on sarcopenia, which added the concept of possible sarcopenia, defined as either low muscle mass or poor physical performance [40]. The cut-off point of grip strength in men was adjusted from 26 to 28 kg, while for women, it was maintained at 18 kg. In addition, the cut-off point of usual gait speed was adjusted from less than 0.8 m/s to 1.0 m/s [29, 40]. Our first participants were recruited in 2018, where, as shown in Table 2, we present a comparison of the diagnoses between the AWGS 2014 and AWGS 2019. The results showed that there was no significant difference in the determination of sarcopenia between the AWGS 2014 and AWGS 2019 in our sample.

No significant differences in the SMI and grip strength were found between the T2DM and control groups; however, gait speed was significantly reduced in patients with T2DM. Leenders et. al [23] reported that patients with diabetes had decreased ASM and grip strength but not in the SMI. A recent study with a larger number of participants showed that although there were no significant differences in the ASM, SMI and grip strength between diabetic patients and normoglycemic controls, a significant decrease in gait speed was found in diabetic patients [41]. These results are similar to our findings. The percentage of occurrences of sarcopenia was higher in the T2DM group, with a ratio of 15.4% compared 6.3% in the control group. This is in accordance with the results in Korean study, in which the prevalence of sarcopenia in patients with diabetes and the control group was 15.7 and 6.9%, respectively [42]. The author speculates that it is due to the similarities in geography and living habits between Taiwan and South Korea. A recent meta-analysis reported that the prevalence of sarcopenia in non-diabetic controls ranged from 8.3% to 16.2% [43]. The prevalence of sarcopenia has also been found to accelerate with increasing age among both genders [44]. The participants in the current study were relatively young, which may have led to the lower frequency of sarcopenia among our participants. In addition, the frequency of possible sarcopenia in the diabetic patients was significantly higher than that in the controls, with 52.6% of the T2DM group and 32% of the controls being in this category. Frailty and depression were found to be significantly higher in the patients with T2DM in the present study, which is in accordance with the findings of previous reports [19, 45–47]. People who meet the ≥ 2/3 criterion in SOF indicate frailty. In this study, there were 14 frail people (17.9%) in the T2DM group (data not shown), which is similar to the results of the recent study [48]. In addition to the STS test, we also measured the strength of the major muscle groups in the upper and lower limbs. We found that STS and muscle strength of the elbow extensor, knee flexor, hip abductor, and hip flexor decreased significantly in the T2DM group. In addition, those muscle groups with decreased muscle strength were mainly considered fast-twitch (type II) muscles [49–51]. The literature indicates that with aging and sarcopenia, type II fibers are more prone to atrophy than type I fibers [52, 53]. We found that the decline in fast-twitch muscle strength predates the decline in muscle mass in patients with diabetes. In previous diabetes research, although the relationship between diabetes and sarcopenia was investigated, it was mainly manifested in the use of magnetic resonance imaging (MRI) or BIA to detect muscle mass, and to evaluate muscle strength and function through grip strength or walking speed test. This research further used a dynamometer to measure the muscle strength of the upper and lower extremities, and found that patients with T2DM have weaker muscle strength in fast-twitch muscles. This may be used as one of the screening indicators for sarcopenia in T2DM. This article suggests that the changes among various muscle groups in diabetic patients can also be used as a reference for physical training in clinical practice.

Nevertheless, we found that the performance of static balance, dynamic balance and aerobic endurance were significantly lower in the patients with T2DM. Åström et al. showed that older people with diabetics exhibit significantly lower overall SFT scores [54]; however, the SFT used in their study mainly focused on muscle strength and flexibility. By contrast, we employed the more comprehensive five-element SFT test for comparisons. We further indicated the impact of T2DM on balance and aerobic endurance. Both static and dynamic balance, which were respectively evaluated by the single-leg-stand test and up-and-go test, were significantly reduced in the diabetic patients. Additionally, aerobic endurance, which was measured by the 2-min step and 6-min walk tests, also showed significant decline in the patients with T2DM.

In this study, there was a significant association between sarcopenia risk-level and T2DM in the univariate analysis; however, the association became attenuated in the multivariate analysis. In the multivariate model, a significant association with T2DM was still observed for BMI, frailty, and dynamic balance. A positive association with T2DM in BMI and frailty has been reported in the previous literature on this topic [55, 56]. This article additionally reports the association of dynamic balance with T2DM. It can be speculated that, at least in this study sample, BMI, frailty, and dynamic balance had stronger associations with T2DM than sarcopenia. The balance limitation in T2DM has been reported to be associated with diabetic peripheral neuropathy [57, 58]. Previous articles have indicated that diabetic neuropathy attenuated the sensorimotor control of the hands and lower extremities [59–61]. However, the pathological mechanism of sarcopenia/frailty in T2DM has been not fully determined. Several possible mechanisms are currently being discussed, including diabetic neuropathy, the decline of neuromuscular junction, hyperglycemia, insulin resistance, chronic inflammation, and mitochondria dysfunction [12, 62]. Based on our findings, we believe that the neurological impact of T2DM affects physical performance, including balance, aerobic endurance, and gait speed, and in turn, these declines subsequently accelerate frailty.

In addition to gait speed, the AWGS 2019 also listed the Short Physical Performance Battery (SPPB) as one of the options for evaluating physical performance [40]. The SPPB assessment contains the chair-stand test, gait-speed test, and balance test. This implies that the importance of balance has been noted in sarcopenia. Previous reports have addressed the association of sarcopenia and frailty with T2DM after the age of 60 years [12, 62, 63]. This study extended the age group to participants over 50 years old and found that there was still a significant difference in the frequency of sarcopenia and frailty between patients with T2DM and the controls. Among the effects on sarcopenia, gait speed represented the most significant difference. We also found that dynamic balance was independently associated with T2DM. This suggests the importance of balance in the early detection of physical deterioration in diabetic patients.

One of the limitations of this study is the size of the population; as such, a larger number of participants is warranted in future research. Another limitation is that this study used the SOF instead of more common frailty tools such as Frailty Index and Clinical Frailty Scale to determine the frailty of the participants. Besides, lack of information regarding the comorbidity and medication status of the recruited participants in this study is also a drawback of this research which may affect some further interpretation regarding the issues of DM-related sarcopenia of older adults.

## Conclusions

This study used more diverse and comprehensive perspectives to investigate the T2DM-related factors, such as SMI, grip strength, gait speed, frailty, depression, upper/lower extremity muscle strength, flexibility, aerobic endurance, static/dynamic balance, etc., to explore the profound manifestations in the patients with T2DM. To sum up, this study indicated the importance of countering frailty and maintaining physical fitness, especially dynamic balance in the early physical deterioration of patients with diabetes. Based on the current findings, further explorations of the effects of different DM severities on different levels of muscle loss as well as influence on different muscle fibre types should be worthy to be investigated. Moreover, the issues regarding exercise trainability or what types of intervention for patients with T2DM to deal with the DM-related sarcopenia might also be a practical topic in the next stage.

## Data Availability

The research group is still working on conducting other analyses based on the current experimental data set used in this study. However, parts of the datasets should be available from the corresponding author after approval by the Institutional Review Board on reasonable request.

## Abbreviations

DM: Diabetes Mellitus
T2DM: Type 2 Diabetes Mellitus
BMI: Body mass index
SFT: Senior fitness test
SMI: Skeletal muscle index
SOF: Study of Osteoporotic Fractures index
FPG: Fasting plasma glucose
OGTT: Oral glucose tolerance test
AWGS: Asian Working Group for Sarcopenia
EWGSOP: European Working Group on Sarcopenia in Older People
SARC-F: Strength, assistance with walking, rising from a chair, climbing stairs, and falls
STS: 30-Second sit-to-stand test
6MWT: 6-Minute walk test
SPSS: Statistical Package for the Social Sciences
OR: Odds ratios
95%CI: 95% Confidence intervals

## References

[1] Saeedi P, Petersohn I, Salpea P, Malanda B, Karuranga S, Unwin N, Colagiuri S, Guariguata L, Motala AA, Ogurtsova K (2019). Global and regional diabetes prevalence estimates for 2019 and projections for 2030 and 2045: Results from the International Diabetes Federation Diabetes Atlas, 9<sup>th</sup> edition. Diabetes Res Clin Pract.

[2] Ramachandran A, Snehalatha C, Shetty AS, Nanditha A (2012). Trends in prevalence of diabetes in Asian countries. World J Diabetes.

[3] Cheng YJ, Imperatore G, Geiss LS, Wang J, Saydah SH, Cowie CC, Gregg EW (2013). Secular changes in the age-specific prevalence of diabetes among U.S. adults: 1988-2010. Diabetes Care.

[4] Ramachandran A, Mary S, Yamuna A, Murugesan N, Snehalatha C (2008). High prevalence of diabetes and cardiovascular risk factors associated with urbanization in India. Diabetes Care.

[5] Sheen YJ, Hsu CC, Jiang YD, Huang CN, Liu JS, Sheu WHH (2019). Trends in prevalence and incidence of diabetes mellitus from 2005 to 2014 in Taiwan. J Formos Med Assoc.

[6] Cruz-Jentoft AJ, Baeyens JP, Bauer JM, Boirie Y, Cederholm T, Landi F, Martin FC, Michel JP, Rolland Y, Schneider SM (2010). Sarcopenia: European consensus on definition and diagnosis: report of the European working group on sarcopenia in older people. Age Ageing.

[7] Janssen I, Heymsfield SB, Wang ZM, Ross R (2000). Skeletal muscle mass and distribution in 468 men and women aged 18-88 yr. J Appl Physiol (Bethesda, Md : 1985).

[8] Sehl ME, Yates FE (2001). Kinetics of human aging: I. Rates of senescence between ages 30 and 70 years in healthy people. J Gerontol A Biol Sci Med Sci.

[9] Shafiee G, Keshtkar A, Soltani A, Ahadi Z, Larijani B, Heshmat R (2017). Prevalence of sarcopenia in the world: a systematic review and meta- analysis of general population studies. J Diabetes Metab Disord.

[10] Trierweiler H, Kisielewicz G, Hoffmann Jonasson T, Rasmussen Petterle R, Aguiar Moreira C (2018). Zeghbi Cochenski Borba V: Sarcopenia: a chronic complication of type 2 diabetes mellitus. Diabetol Metab Syndr.

[11] Souza ABF, Nascimento DAC, Rodrigues IJM, Charone CCO, Lopes GL, Lima RS, Sa AA, Carneiro TX, Moraes NS (2019). Association between sarcopenia and diabetes in community dwelling elderly in the Amazon region - Viver Mais Project. Arch Gerontol Geriatr.

[12] Umegaki H (2016). Sarcopenia and frailty in older patients with diabetes mellitus. Geriatr Gerontol Int.

[13] Assar ME, Laosa O, Rodríguez Mañas L (2019). Diabetes and frailty. Curr Opin Clin Nutr Metab Care.

[14] Perkisas S, Vandewoude M (2016). Where frailty meets diabetes. Diabetes Metab Res Rev.

[15] Morley JE, Vellas B, van Kan GA, Anker SD, Bauer JM, Bernabei R, Cesari M, Chumlea WC, Doehner W, Evans J (2013). Frailty consensus: a call to action. J Am Med Dir Assoc.

[16] Yanase T, Yanagita I, Muta K, Nawata H (2018). Frailty in elderly diabetes patients. Endocr J.

[17] Sinclair AJ, Conroy SP, Bayer AJ (2008). Impact of diabetes on physical function in older people. Diabetes Care.

[18] Sayer AA, Dennison EM, Syddall HE, Gilbody HJ, Phillips DIW, Cooper C (2005). Type 2 diabetes, muscle strength, and impaired physical function. Diabetes Care.

[19] Wong E, Backholer K, Gearon E, Harding J, Freak-Poli R, Stevenson C, Peeters A (2013). Diabetes and risk of physical disability in adults: a systematic review and meta-analysis. Lancet Diabetes Endocrinol.

[20] Martinez-Huedo MA, Lopez de Andres A, Hernandez-Barrera V, Palacios-Ceña D, Carrasco-Garrido P, Hernandez DM, Jiménez-Garcia R (2011). Trends in the prevalence of physical and functional disability among Spanish elderly suffering from diabetes. Diabetes Res Clin Pract.

[21] McLaughlin D, Leung J, Byles J, Dobson A (2011). Living with stairs: functioning in a large cohort of older Australian adults. J Am Geriatr Soc.

[22] Odding E, Valkenburg HA, Stam HJ, Hofman A (2001). Determinants of locomotor disability in people aged 55 years and over: the Rotterdam Study. Eur J Epidemiol.

[23] Leenders M, Verdijk LB, van der Hoeven L, Adam JJ, van Kranenburg J, Nilwik R, van Loon LJ (2013). Patients with type 2 diabetes show a greater decline in muscle mass, muscle strength, and functional capacity with aging. J Am Med Dir Assoc.

[24] Gregg EW, Beckles GL, Williamson DF, Leveille SG, Langlois JA, Engelgau MM, Narayan KM (2000). Diabetes and physical disability among older U.S. adults. Diabetes Care.

[25] Volpato S, Ferrucci L, Blaum C, Ostir G, Cappola A, Fried LP, Fellin R, Guralnik JM (2003). Progression of lower-extremity disability in older women with diabetes: the Women's Health and Aging Study. Diabetes Care.

[26] Bandinelli S, Benvenuti E, Del Lungo I, Baccini M, Benvenuti F, Di Iorio A, Ferrucci L (1999). Measuring muscular strength of the lower limbs by hand-held dynamometer: a standard protocol. Aging (Milan, Italy).

[27] Jones CJ, Rikli RE (2002). Measuring Functional Fitness in Older Adults. The Journal of Active Ageing..

[28] Chamberlain JJ, Rhinehart AS, Shaefer CF, Neuman A (2016). Diagnosis and management of diabetes: synopsis of the 2016 American diabetes association standards of medical care in diabetes. Ann Intern Med.

[29] Chen LK, Liu LK, Woo J, Assantachai P, Auyeung TW, Bahyah KS, Chou MY, Chen LY, Hsu PS, Krairit O (2014). Sarcopenia in Asia: consensus report of the Asian Working Group for Sarcopenia. J Am Med Dir Assoc.

[30] Malmstrom TK, Morley JE (2013). SARC-F: a simple questionnaire to rapidly diagnose sarcopenia. J Am Med Dir Assoc.

[31] Ensrud KE, Ewing SK, Taylor BC, Fink HA, Stone KL, Cauley JA, Tracy JK, Hochberg MC, Rodondi N, Cawthon PM (2007). Frailty and risk of falls, fracture, and mortality in older women: the study of osteoporotic fractures. J Gerontol A Biol Sci Med Sci.

[32] Ensrud KE, Ewing SK, Cawthon PM, Fink HA, Taylor BC, Cauley JA, Dam TT, Marshall LM, Orwoll ES, Cummings SR (2009). A comparison of frailty indexes for the prediction of falls, disability, fractures, and mortality in older men. J Am Geriatr Soc.

[33] Hu B, Hsiao-Wei Y, Chiu T, Li-Ling L, Chen Y (2018). The validity of the Study Of Osteoporotic Fractures (SOF) index for assessing community-based older adults in Taiwan. Innov Aging.

[34] Maurer DM (2012). Screening for depression. Am Fam Physician.

[35] Rikli RE, Jones CJ (2013). Development and validation of criterion-referenced clinically relevant fitness standards for maintaining physical independence in later years. Gerontologist.

[36] Hesseberg K, Bentzen H, Bergland A (2015). Reliability of the senior fitness test in community-dwelling older people with cognitive impairment. Physiother Res Int.

[37] Lin PS, Hsieh CC, Cheng HS, Tseng TJ, Su SC (2016). Association between physical fitness and successful aging in Taiwanese older adults. PloS One.

[38] Chen HT, Lin CH, Yu LH (2009). Normative physical fitness scores for community-dwelling older adults. J Nurs Res.

[39] Bohannon RW (1997). Reference values for extremity muscle strength obtained by hand-held dynamometry from adults aged 20 to 79 years. Arch Phys Med Rehabil.

[40] Chen LK, Woo J, Assantachai P, Auyeung TW, Chou MY, Iijima K, Jang HC, Kang L, Kim M, Kim S (2020). Asian working group for sarcopenia: 2019 consensus update on sarcopenia diagnosis and treatment. J Am Med Dir Assoc.

[41] Wang T, Feng X, Zhou J, Gong H, Xia S, Wei Q, Hu X, Tao R, Li L, Qian F (2016). Type 2 diabetes mellitus is associated with increased risks of sarcopenia and pre-sarcopenia in Chinese elderly. Sci Rep.

[42] Kim TN, Park MS, Yang SJ, Yoo HJ, Kang HJ, Song W, Seo JA, Kim SG, Kim NH, Baik SH (2010). Prevalence and determinant factors of sarcopenia in patients with type 2 diabetes: the Korean Sarcopenic Obesity Study (KSOS). Diabetes Care.

[43] Pacifico J, Geerlings MAJ, Reijnierse EM, Phassouliotis C, Lim WK, Maier AB (2020). Prevalence of sarcopenia as a comorbid disease: A systematic review and meta-analysis. Exp Gerontol.

[44] Khongsri N, Tongsuntud S, Limampai P, Kuptniratsaikul V (2016). The prevalence of sarcopenia and related factors in a community-dwelling elders Thai population. Osteoporosis and Sarcopenia.

[45] Hubbard RE, Andrew MK, Fallah N, Rockwood K (2010). Comparison of the prognostic importance of diagnosed diabetes, co-morbidity and frailty in older people. Diabet Med.

[46] Anderson RJ, Freedland KE, Clouse RE, Lustman PJ (2001). The prevalence of comorbid depression in adults with diabetes: a meta-analysis. Diabetes Care.

[47] Park M, Reynolds CF (2015). 3rd: Depression among older adults with diabetes mellitus. Clin Geriatr Med.

[48] Chhetri JK, Zheng Z, Xu X, Ma C, Chan P (2017). The prevalence and incidence of frailty in Pre-diabetic and diabetic community-dwelling older population: results from Beijing longitudinal study of aging II (BLSA-II). BMC Geriatr.

[49] Garrett WE, Califf JC, Bassett FH (1984). Histochemical correlates of hamstring injuries. Am J Sports Med.

[50] Arbanas J, Klasan GS, Nikolic M, Jerkovic R, Miljanovic I, Malnar D (2009). Fibre type composition of the human psoas major muscle with regard to the level of its origin. J Anat.

[51] Kovanen V, Suominen H, Heikkinen E (1984). Collagen of slow twitch and fast twitch muscle fibres in different types of rat skeletal muscle. Eur J Appl Physiol.

[52] Narici MV, Maffulli N (2010). Sarcopenia: characteristics, mechanisms and functional significance. Br Med Bull.

[53] Marzetti E, Lees HA, Wohlgemuth SE, Leeuwenburgh C (2009). Sarcopenia of aging: underlying cellular mechanisms and protection by calorie restriction. BioFactors (Oxford, England).

[54] Åström MJ, von Bonsdorff MB, Perälä MM, Salonen MK, Rantanen T, Kajantie E, Simonen M, Pohjolainen P, Osmond C, Eriksson JG (2018). Glucose regulation and physical performance among older people: the Helsinki Birth Cohort Study. Acta Diabetol.

[55] Bays HE, Chapman RH, Grandy S, Group SI (2007). The relationship of body mass index to diabetes mellitus, hypertension and dyslipidaemia: comparison of data from two national surveys. Int J Clin Pract.

[56] Lee JS, Auyeung TW, Leung J, Kwok T, Leung PC, Woo J (2011). Physical frailty in older adults is associated with metabolic and atherosclerotic risk factors and cognitive impairment independent of muscle mass. J Nutr Health Aging.

[57] Corriveau H, Prince F, Hébert R, Raîche M, Tessier D, Maheux P, Ardilouze JL (2000). Evaluation of postural stability in elderly with diabetic neuropathy. Diabetes Care.

[58] Jernigan SD, Pohl PS, Mahnken JD, Kluding PM (2012). Diagnostic accuracy of fall risk assessment tools in people with diabetic peripheral neuropathy. Phys Ther.

[59] Chiu H-Y, Hsu H-Y, Kuo L-C, Su F-C, Yu H-I, Hua S-C, Lu C-H (2014). How the impact of median neuropathy on sensorimotor control capability of hands for diabetes: an achievable assessment from functional perspectives. PLoS ONE.

[60] Hsu H-Y, Chiu H-Y, Lin H-T, Su F-C, Lu C-H, Kuo L-C (2015). Impacts of elevated glycaemic haemoglobin and disease duration on the sensorimotor control of hands in diabetes patients. Diabetes Metab Res Rev.

[61] Boulton AJ, Vinik AI, Arezzo JC, Bril V, Feldman EL, Freeman R, Malik RA, Maser RE, Sosenko JM, Ziegler D (2005). Diabetic neuropathies: a statement by the American Diabetes Association. Diabetes Care.

[62] Sinclair AJ, Abdelhafiz AH, Rodríguez-Mañas L (2017). Frailty and sarcopenia - newly emerging and high impact complications of diabetes. J Diabetes Complications.

[63] Morley JE, Malmstrom TK, Rodriguez-Mañas L, Sinclair AJ (2014). Frailty, Sarcopenia and Diabetes. J Am Med Dir Assoc.

